# Molecular genetics of phenylketonuria and tetrahydrobiopterin deficiency in Jordan

**DOI:** 10.1002/jmd2.12130

**Published:** 2020-05-19

**Authors:** Carla Carducci, Wajdi Amayreh, Haneen Ababneh, Amjad Mahasneh, Buthaina Al Rababah, Kefah Al Qaqa, Momen Al Aqeel, Cristiana Artiola, Manuela Tolve, Sirio D'Amici, Nan Shen, Yongguo Yu, Alicia Hillert, Nastassja Himmelreich, Jürgen G. Okun, Georg F. Hoffmann, Nenad Blau

**Affiliations:** ^1^ Department of Experimental Medicine University of Rome “La Sapienza” Rome Italy; ^2^ Queen Rania Children Hospital, King Hussein Medical Centre Amman Jordan; ^3^ Princess Haya Biotechnology Centre Jordan University of Science and Technology Irbid Jordan; ^4^ Department of Biotechnology and Genetic Engineering Jordan University of Science and Technology Irbid Jordan; ^5^ Department of Rehabilitation Medicine Xin Hua Hospital affiliated to Shanghai Jiao Tong University School of Medicine Shanghai China; ^6^ Department of Pediatric Endocrinology and Genetic Metabolism Shanghai Institute for Pediatric Research, Xinhua Hospital, School of Medicine, Shanghai Jiao Tong University Shanghai China; ^7^ Dietmar Hopp Metabolic Center and Centre for Pediatrics and Adolescent Medicine University Hospital Heidelberg Heidelberg Germany; ^8^ Division of Metabolism University Children's Hospital Zürich Switzerland

**Keywords:** genotyping, newborn screening, phenylketonuria, PKU incidence, tetrahydrobiopterin

## Abstract

**Background:**

Information regarding the prevalence of PKU in the Middle East in comparison to other world regions is scarce, which might be explained by difficulties in the implementation of national newborn screening programs.

**Objective:**

This study seeks for the first time to genotype and biochemically characterize patients diagnosed with hyperphenylalaninemia (HPA) at the Pediatric Metabolic Genetics Clinic at the King Hussein Medical Center, Amman, Jordan.

**Methods:**

A total of 33 patients with HPA and 55 family members were investigated for pterins (neopterin and biopterin) and dihydropteridine reductase (DHPR) activity in dried blood spots. Patients with HPA were genotyped for phenylketonuria (PKU) and the genes involved in tetrahydrobiopterin (BH_4_) metabolism.

**Results:**

In total 20 patients were diagnosed with PKU due to phenylalanine hydroxylase (PAH) deficiency, 2 with GTP cyclohydrolase I (GTPCH) deficiency, 6 with DHPR deficiency, and 3 with the 6‐pyruvoyl‐tetrahydropterin synthase (PTPS) deficiency. Diagnosis was not possible in 2 patients. This study documents a high percentage of BH_4_ deficiencies within HPA patients. With one exception, all patients were homozygous for particular gene variants.

**Conclusions:**

This approach enables differentiation between PKU and BH_4_ deficiencies and, thus, allows for critical selection of a specific treatment strategies.

**SYNOPSIS**

The paper describes for the first time genotypes and phenotypes of PKU patients and a relative high incidence of BH4 deficiencies in Jordan.

## 1 INTRODUCTION

Phenylketonuria (PKU; OMIM# 261600) is an autosomal recessive metabolic disease caused by deficiency of hepatic phenylalanine‐4‐hydroxylase (PAH; EC 1.14.16.1).^1^ PAH is a non‐heme iron enzyme that catalyses the rate‐limiting step in phenylalanine (Phe) catabolism using molecular oxygen, iron and tetrahydrobiopterin (BH_4_) as cofactors.^2^ If untreated, biallelic loss of function variants in the *PAH* gene therefore lead to hyperphenylalaninemia (HPA) and accumulation of Phe in the brain, causing severe intellectual disability, behavioural disturbances and psychiatric disorders in untreated patients.^3^ While introduction of a Phe‐restricted diet in the first 2 to 3 weeks has been shown to nearly abolish these symptoms, some patients treated from an early age show lower IQ scores than their matched controls, while cognitive outcomes are inversely correlated with blood Phe levels.^4^ The clear relationship between clinical and metabolic phenotypes is the basis of phenotype classification, which is based on blood Phe levels before starting the treatment.^5^, ^6^ Most classifications today distinguish between mild hyperphenylalaninemia (MHP; 120‐600 μmol/L), mild PKU (mPKU; 600‐1200 μmol/L) and classic PKU (cPKU; >1200 μmol/L), while some recognize an additional group of moderate PKU (blood Phe 900‐1200 μmol/L). The wide range of metabolic (and clinical) phenotypes has been shown to be mainly determined by the *PAH* genotype although other factors might play a role as well.^7^ The large PKU database BIOPKU (http://www.biopku.org) enables the genotypic phenotype prediction in almost 99% of patients by the using allelic phenotype values (APVs).^8^

PKU is the most common inborn error of amino acid metabolism, affecting approximately 1 in 10 000 newborns in Europe.^1^ The prevalence of PKU ranges from rather rare (e.g., in Japan 1:120000 and Thailand 1:122000), to very common (e.g., in the Karachay‐Cherkess Republic of Russia 1:850^9^ and Sicily, Italy 1:2700).^10^ Prevalence reports in the Arab countries are scarce,^11^ and there is no solid data on the prevalence of PKU in Jordan.^12^, ^13^

In this study, we characterized patients with HPA and their family members from the Pediatric Metabolic Genetics Clinic at the King Hussein Medical Center, Amman, Jordan biochemically and genetically, differentiating between PKU and BH_4_ deficiencies.

## 2 MATERIALS AND METHODS

### 2.1 Patients

A total of 33 patients (13 females and 20 males; age 1 week to 1 year and 8 months) from 25 families of Jordanian background (East Jordan and west bank that is Palestine) were diagnosed with HPA (blood Phe levels 205‐2107 μmol/L) at the Pediatric Metabolic Genetics Clinic at King Hussein Medical Center, Amman, Jordan (Table 1). Eleven clinically normal patients were diagnosed through the newborn screening for PKU before the age of 1 week, while all other patients were diagnosed late. They presented with developmental delay, hypotonia, spasticity, hyperreflexia microcephaly, seizures, ADHS or abnormal brain MRI at the time of investigations (Table 1). Consanguinity (first or second cousins) was reported in 19 (57%) patients.

**TABLE 1:** Patients information at the diagnosis and at the time of investigations

ID No.	Gender	Consanguinity	Age at diagnosis	Phe (μmol/L)	Age at investigations	Microcephaly	Hypotonia	Epilepsy	Developmental delay	ADHS	Brain MRI abnormal	Spasticity	Hyperreflexia	Died
1	M	Second cousin	10 m	1670	3 y 4 mo	+	+	+	+		+			
2	F	First cousin	1 wk	354	1 y 8 mo									
3	F	First cousin	1 y 6 mo	755	6 y 5 mo	+			+	+		+	+	
4	F	Second cousin	2 wk	777	1 y 5 mo	+	+		+					
5	F	−	1 wk	1300	2 y									
6	F	Second cousin	2 mo	785	3 y 5 mo					+				
7	M	First cousin	1 y 6 mo	−	5 y 11 mo	+	+		+	+				
8	F	−	2 mo	926	1 y 4 mo									
9	M	First cousin	1 wk	1240	2 y 2 mo									
10	M	First cousin	1 wk	1150	2 y									
11	M	−	1 wk	908	8 m									
12	F	First cousin	1 wk	1301	1 y 5 mo									
13	F	First cousin	1 y 6 mo	1152	20 y 9 mo	+	+	+	+		+			
14	M	Not related	1 mo	1100	3 y 6 mo	+		+	+		+	+	+	
15	F	Not related	1 mo	400	5 y 11 mo									
16	M	First cousin	1 y 6 mo	−	21 y 9 mo	+		+	+		+			
17	M	−	1 wk	−	13 y 2 mo									
18	M	−	1 wk	−	13 y 2 mo									
19	M	−	1 wk	−	13 y 2 mo									
20	M	Second cousin	1 y 6 mo	−	11 y 2 mo	+			+			+		
21	M	First cousin	−	−	8 y 5 mo	+			+		+	+	+	
22	F	First cousin	1 y 6 mo	−	7 y 9 mo		+		+					
23	M	−	1 y 8 mo	288	7 y 1 mo	+		+	+++		+	+	+	
24	M		8 mo	205	2 y 7 mo	+			++					
25	F	Not related	9 mo	−	6 y 11 mo	+		+	+++		+	+	+	
26	M	First cousin	7 mo	−	4 y 4 mo		+	+	++	+	+	+	+	
27	M	−	−	−	8 y 3 mo	+		+	+++	+	+	+	+	
28	M	−	−	−	10 y 5mo	+		+	+++	+	+	+	+	
29	M	First cousin	1 wk	1016	1 y 3 mo									+
30	F	First cousin	3 mo	2107	1 y 5 mo	+	+	+	+			+	+	
31	M	First cousin	1 mo	918	1 y 1 mo	+	+	+	+			+	+	
32	M	Not related	1 wk	1431	1 y 6 mo	+	+						+	
33	F	First cousin	4 mo	347	1 y 5 mo	+	+	+	+		+	+	+	+

Abbreviation: ADSH, attention deficit hyperactivity syndrome.

### 2.2 Pterins and DHPR testing

With one exception (#18), all patients (n = 32) and their parents (n = 55) were investigated for pterins (neopterin and biopterin) and dihydropteridine reductase (DHPR) activity in dried blood spots (DBSs). All patients with HPA were genotyped for variations in *PAH, GCH1* (GTP cyclohydrolase 1)*, PTS* (6‐pyruvoly‐tetrahydropterin synthase) and *QDPR* (DHPR) genes to exclude or confirm a possible BH_4_ deficiency. Pterins were measured in DBSs using the method described by Opladen et al,^14^ and DHPR activity was measured according to Arai et al.^15^

### 2.3 DNA sequencing

DNA testing was carried out by Sanger sequencing of the CDS regions and the exon‐intron boundaries of the four genes, following biochemical findings. For two patients, the GTPCH feed‐back regulatory protein (*GFRP)* and *DNAJC12* genes were also investigated. The primers and PCR conditions used were original and are available upon request. All sequencing reactions were performed using a BigDyeTerminator Cycle Sequencing v1.1 kit (ThermoFisher) and analyzed on an ABI PRISM 3130 XL (ThermoFisher). To investigate *PAH* exon deletion/duplication a MLPA kit was used (MRC‐Holland) according to the manufacturer's recommendations.

APV is a value defining the association of a variant with the corresponding metabolic phenotype, thus defining its severity. APV was calculated for variants occurring in a functionally hemizygous constellation (ie, in a combination with an inactive zero‐allele).^8^ APVs range between 0 and 10, with following classification cPKU (APV = 0‐2.7), mPKU (APV = 2.8‐6.6), and MHP (APV = 6.7‐10).

Whole exome sequencing was performed in two patients for which no mutations were found in the *PAH*, *GCH1*, *PTS*, *QDPR*, *GFRP* or *DNAJC12* genes. Genomic DNA of probands and their family members was extracted from peripheral blood leukocytes using a Lab‐Aid Nucleic Acid Isolation kit (Zeesan, China), according to the manufacturer's instructions. Exome sequencing was performed on genomic DNA. Capture was conducted with the xGen Exome research panel v1.0 (Integrated DNA Technologies), and the captured DNA fragments were sequenced by a HiSeq 4000 (Illumina). A Burrows‐Wheeler Aligner (BWA, version 0.7.10) was used to map reads to the human reference genome (GRCh37/hg19). Base calling, QC analysis and coverage analysis were performed with Picard tools 1.124 and GATK software. Variants were annotated using SnpEff version 4.2. Subsequently, the following variants were filtered out: (a) variants with >1% frequency in the population variant databases, including the 1000 Genomes Project, Exome Variant Server (EVS) and Exome Aggregation Consortium (ExAC), or >5% frequency in our in‐house database (based on 150 exome datasets), (b) intergenic and 3′/5′ untranslated region variants, non‐splice‐related intronic and synonymous variants. MutationTaster (http://www.mutationtaster.org), SIFT (http://sift.jcvi.org), and PolyPhen‐2 (http://genetics.bwh.harvard.edu/pph2/) were used to assess the pathogenic potential of the variants. Combined with clinical manifestation and modes of inheritance, candidate variants were validated by Sanger sequencing and classified according to standards and guidelines of the American College of Medical Genetics and Genomics (ACMG). Parents were not studied.

This study was approved by the local ethics commission. Written informed consent was obtained from each adult patient or from the parent or guardian of each child, adolescent and intellectually disabled patient enrolled in this study.

## 3 RESULTS

Out of all 33 investigated patients from Jordan, 20 patients were classified as having PKU caused by PAH deficiency (18 cPKU and 2 mPKU), and 11 were diagnosed with BH_4_ deficiencies (6 with DHPR deficiency, 3 with PTPS deficiency and 2 with GTPCH deficiency). For two patients, no variations were found in *PAH*, *GCH1*, *PTS*, *QDPR*, *GFRP*, or *DNAJC12*(Table 2).

**TABLE 2:** Pterins and DHPR activity in dried blood spots and genotypes of 33 HPA patients from Jordan

No.	Age at investigations	Neo (DBS)	Bio (DBS)	%Bio (Bio/(Neo+Bio)*100)	DHPR (DBS)	Gene	Protein variant	Genotype	GPV^a^	PolyPhen2/SIFT^b^	Phenotype
		nmol/g Hb									
1	3 y 4 mo	0.39	0.22	36	1.7	*PAH*	p.[Arg176*]; [Arg176*]	c.[526C>T]; [526C>T]	0	‐	cPKU
2	1 y 8 mo	0.40	0.22	35	1.0	*PAH*	p.[Leu197Phe]; [Leu197Phe]	c.[591G>C]; [591G>C]	3.8^c^	1.000/0.01	mPKU
3	6 y 5 mo	0.72	0.45	38	2.0	*PAH*	p.[Phe55Leufs*6]; [Phe55Leufs*6]	c.[165del]; [165del]	0	‐	cPKU
4	1 y 5 mo	1.34	0.38	22	2.1	*PAH*	p.[Arg261Gln]; [Arg261Gln]	c.[782G>A]; [782G>A]	1.6	1.00/00.01	cPKU
5	2 y	0.88	0.34	28	1.2	*PAH*	p.(?)	c.[1066‐11G>A]; [1066‐11G>A]	0	‐	cPKU
6	3 y 5 mo	0.59	0.70	54	0.9	*PAH*	p.[Arg176*]; [Arg176*]	c.[526C>T]; [526C>T]	0	‐	cPKU
7	5 y 11 mo	0.74	0.48	39	1.3	*PAH*	p.[Phe55Leufs*6]; [Phe55Leufs*6]	c.[165del]; [165del]	0	‐	cPKU
8	1 y 4 mo	0.74	0.21	22	1.7	*PAH*	p.[Phe55Leufs*6]; [Phe55Leufs*6]	c.[165del]; [165del]	0	‐	cPKU
9	2 y 2 mo	0.27	0.09	25	2.8	*PAH*	p.[Arg176*]; [Arg176*]	c.[526C>T]; [526C>T]	0	‐	cPKU
10	2 y	0.32	0.11	26	1.2	*PAH*	p.[Thr323del]; [Thr323del]	c.[967_969del]; [967_969del]	0	‐	cPKU
11	8 mo	1.36	0.25	16	2.7	*PAH*	p.[Thr323del]; [Thr323del]	c.[967_969del]; [967_969del]	0	‐	cPKU
12	1 y 5 mo	0.64	0.44	41	1.7	*PAH*	p.[Lys363Asnfs*37]; [Lys363Asnfs*37]	c.[1089del]; [1089del]	0	‐	cPKU
13	20 y 9 mo	0.59	0.35	37	2.7	*PAH*	p.[Glu57del]; [Glu57del]	c.[169_171del]; [169_171del]	0	‐	cPKU
14	3 y 6 mo	0.43	0.24	36	3	*PAH*	p.[Arg176*]; [Arg176*]	c.[526C>T]; [526C>T]	0	‐	cPKU
15	5 y 11 mo	0.67	0.24	26	1.6	*PAH*	p.[Arg261Gln]; [Leu197Phe]	c.[782G>A]; [591G>C]	3.8^c^	1.000/0.01	mPKU
16	21 y 9 mo	0.19	0.24	56	1.7	*PAH*	p.[Tyr198Serfs*136]; [Tyr198Serfs*136]	c.[592_613del]; [592_613del]	0	‐	cPKU
17	13 y 2 mo	0.52	0.35	40	1.9	*PAH*	p.[Tyr198Serfs*136]; [Tyr198Serfs*136]	c.[592_613del]; [592_613del]	0	‐	cPKU
18	13 y 2 mo	‐	‐	‐	‐	*PAH*	p.[Tyr198Serfs*136]; [Tyr198Serfs*136]	c.[592_613del]; [592_613del]	0	‐	cPKU
19	13 y 2 mo	0.22	0.11	33	2	*PAH*	p.[Tyr198Serfs*136]; [Tyr198Serfs*136]	c.[592_613del]; [592_613del]	0	‐	cPKU
20	11 y 2 mo	0.2	0.08	29	3	*PAH*	p.(?)	c.[1199+1G>C]; [1199+1G>C]	0	‐	cPKU
21	8 y 5 mo	0.14	0.01	7	2.4	*GCH1*	p.[His126Leu]; [His126Leu]^d^	c.[377A>T]; [377A>T]		0.131/0.01	GTPCH
22	7 y 9 mo	0.03	0.03	40	2.8	*GCH1*	p.[His126Leu]; [His126Leu]^d^	c.[377A>T]; [377A>T]		0.131/0.01	GTPCH
23	7 y 1 mo	0.48	0.18	27	<0,1	*QDPR*	p.[Gly170Ser]; [Gly170Ser]	c.[508G>A]; [508G>A]		0.999/0.00	DHPR
24	2 y 7 mo	0.88	0.16	15	<0,1	*QDPR*	p.[Gly170Ser]; [Gly170Ser]	c.[508G>A]; [508G>A]		0.999/0.00	DHPR
25	6 y 11 mo	0.68	0.26	28	<0,1	*QDPR*	p.(?)	c.[436+1G>C]; [436+1G>C]		‐	DHPR
26	4 y 4 mo	1	0.99	50	<0,1	*QDPR*	p.(?)	c.[545+1G>A]; [545+1G>A]		‐	DHPR
27	8 y 3 mo	0.59	1.36	70	<0,1	*QDPR*	p.(?)	c.[545+1G>A]; [545+1G>A]		‐	DHPR
28	10 y 5 mo	0.57	1.32	70	<0,1	*QDPR*	p.(?)	c.[545+1G>A]; [545+1G>A]		‐	DHPR
29	1 y 3 mo	4.44	0.01	0	3.4	*PTS*	p.[Thr67Met]; [Thr67Met]	c.[200C>T]; [200C>T]		1.000/0.01	PTPS
30	1 y 5 mo	9.4	0.01	0	2.2	*PTS*	p.[Asp94Gly]; [Asp94Gly]^d^	c.[281A>G]; [281A>G]		0.875/0.05	PTPS
31	1 y 1 mo	1.63	0.02	1	2.4	*PTS*	p.[Glu82Gly]; [Glu82Gly]	c.[245A>G]; [245A>G]		0.024/0.05	PTPS
32	1 y 6 mo	1.06	0.39	27	2.7	‐					HPA
33	1 y 5 mo	1.26	0.31	20	2.3	‐					HPA
	Controls	0.2‐2.9	0.1‐1.2		>1.4						

a GPV (=APVmax): genotypic phenotype value (0‐2.7 cPKU, 2.8‐6.6 mPKU, 6.7‐10 MHP).

b PolyPhen2 (0‐0.15 = benign, 0.15‐1.0 = possibly damaging, 0.85‐1.0 = damaging), SIFT (0‐0.05 = deleterious, 0.05‐1.0 = tolerated/benign).

c Based on phenotypes of only four patients.

d Variants previously not reported. Neo, neopterin; Bio, biopterin; HPA, hyperphenylalaninemia; DHPR, dihydropteridine reductase; *PAH* phenylalanine hydroxylase gene (NM_000277.3); *GCH1*, GTP cyclohydrolase I gene (NM_000161.3); GTPCH, GTP cyclohydrolase I; *QDPR*, dihydropteridine reductase gene (NM_000320.3); DHPR, dihydropteridine reductase; *PTS*, 6‐pyruvoy‐tetrahydropterine synthase gene (NM_000317.3); PTPS, 6‐pyruvoy‐tetrahydropterine synthase; cPKU, classic PKU; mPKU, mild PKU; MHP, mild hyperphenylalaninemia.

Out of the 20 PAH‐deficient PKU patients, 19 were homozygous and only 1 was compound heterozygote (p.[Arg261Gln];[Leu197Phe]). Ten different variants could be identified (c.1066‐11G>A, c.1199+1G>C, p.Glu57del, p.Lys363Asnfs*37, p.Leu197Phe, p.Arg261Gln, p.Thr323del, p.Arg176*, p.Phe55Leufs*6, p.Tyr198Serfs*136), among which p.Tyr198Serfs*136 and p.Arg176* (each with 20%) were the most frequent, followed by p.Phe55Leufs*6 with 15%. All variants had an APV between 0 and 1.3, indicating classical PKU. The exception was p.Leu197Phe (mPKU), where the APV value was unknown. The pterins pattern (neopterin and biopterin) in DBS was inconspicuous in all PKU patients (Table 2).

Two patients diagnosed with the GTPCH deficiency carried the same not previously described homozygous *GCH1* variant (p.His126Leu), and their DBS neopterin and biopterin levels were very low (Table 2).

The patients with DHPR deficiency were all homozygous for the following *QDPR* variants: p. Gly170Ser, c.436+1G>C and c.545+1G>A. Interestingly, all these patients had normal pterin patterns, with the exception of the elevated DBS biopterin level in patients #26 and #27. Furthermore, all patients in this subgroup were severely affected by a developmental delay and one patient (#29) passed away (Table 2).

The 3 PTPS‐deficient patients were all children of first degree cousin marriages, which might explain the homozygous constellation of the *PTS* variants p.Thr67Met, p.Asp94Gly (new), and p.Glu82Gly in these patients (Table 2). The p.Glu82Gly variants was predicted by SIFT algorithm to be tolerated (benign), but the patient (#31) present with neurological problems similar to other PTPS‐deficient patients.

In all family members, the DBS pterins pattern and DHPR activity were normal (data not shown).

The DNA of two patients with a normal pterins pattern in DBS and with no mutation detected in the *PAH, GCH1, PTS, QDPR, GFRP* and *DNAJC12* genes underwent whole exome sequencing, but again with no clear diagnosis. In one patient who died (#33), a heterozygous *GCH1* variant of unknown significance (c.*721A>G) was found in the 3'UTR region downstream of the stop codon.

## 4 DISCUSSION

Unfortunately, little information is available regarding the prevalence of PKU in the Middle East in comparison to other world regions, which might be explained by difficulties in the implementation of national newborn screening (NBS) programs.^16^ Only 10 Middle Eastern countries, including Bahrain, Egypt, Iran, Israel, Kuwait, Oman, Qatar, the State of Palestine, Saudi Arabia, and the United Arab Emirates, have extensive national NBS programs.^17^ However, the obtainable data suggest that certain genetic disorders, such as PKU, occur more often in Arabic than in Western countries, which can be explained by several factors. First, the typical large Arabic family structure becomes apparent if one compares the crude birth rate of the Middle East and North Africa (MENA) regions—23,1 per 1000 population (over 10,15 million births in 2017)—with the crude birth rate in the United States of 11,8 per 1000 (3,8 million births in 2017).^18^

The incidence of PKU and other inherited metabolic disorders in Jordan is not known, although the incidence of inherited metabolic disorders is 1:1327 in Qatar, 1:1381 in Saudi Arabia, and 1:1555 in Oman, which could represent the type of population in Jordan, with high rates of consanguinity.^12^ In 2006, the first PKU screening project was started in certain areas of Jordan, and since 2010 the Ministry of Health expanded it to a nationwide screening program and provides care for the detected cases. By 2012, a total of 168 PKU patients were diagnosed, in which only 32% were detected in the newborn period, indicating that screening still needs to be improved. The estimated incidence of PKU in Jordan is approximately 1:10 000 live births, and one of the main factors contributing to this high occurrence of PKU is the high degree of consanguineous marriages.^13^

Our study demonstrates a predominantly severe classic PKU phenotype in Jordanian patients, with two exceptions (mPKU), all carrying homozygous mutations with no residual PAH activity. Out of 10 *PAH* variants detected, 2 were missense variants (p.Arg261Gln and p.Leu197Phe with residual enzyme activity), 3 were frame shifts, 2 were in‐frame deletions, 2 were splice variants, and one was a nonsense variant (Table 2). In all cases, the metabolic phenotype fitted well with the observed genotype and with the calculated genotypic phenotype value (GPV; equal with the higher APV of the two alleles), confirming the power of genotypic phenotype prediction in PKU.^8^ All PKU patients adhered to a low‐Phe diet and their condition was under good control; 6 late‐diagnosed patients were on anticonvulsants and presented with psychomotor retardation, developmental delay and seizures.

Disorders of BH_4_ metabolism account for only 1% to 2% of patients with HPA in Europeans,^19^ whereas they are more common in some other ethnic groups. For example, BH_4_ deficiency accounts for more than 10% of all HPA patients in some countries in East Asia, including Taiwan, Mainland China, Japan, South Korea, the Philippines, Thailand and Malaysia,^20^ reaching up to one‐third of cases in some reports.^21^ The current study also demonstrates a relatively frequent occurrence of BH_4_ deficiency in Jordan. A total of 11 (33%) out of 33 patients with HPA were diagnosed with BH_4_ deficiency (6 DHPR, 3 PTPS, and 2 GTPCH). These findings align with previous reports from other countries in the region. A high incidence of BH_4_ deficiency (~12%) was reported for the Kingdom of Saudi Arabia^22^ and Iran.^23^

Given the high prevalence of BH_4_ deficiency in Arab countries and the fact that early diagnosis and treatment can prevent brain damage, a systematic investigation of BH_4_ deficiency is essential for all newborns and infants with HPA. Simple tests for pterin and DHPR activity in DBS enable the diagnosis of all forms of BH_4_ deficiency presenting with HPA. With an expanded NBS program and the wide availability of next‐generation sequencing methods, diagnostic testing will become available for the region.

## CONFLICT OF INTEREST

The authors declare no conflicts of interest.

## AUTHOR CONTRIBUTIONS

Carla Carducci, Wajdi Amayreh and Nenad Blau planned and designed the study, performed the analysis, evaluated study results, participated in drafting and revising of the manuscript and read and approved the final version before submission. Haneen Ababneh, Amjad Mahasneh, Buthaina Al Rababah, Kefah Al Qaqa, Momen Al Aqeel, Cristiana Artiola, Manuela Tolve, Sirio D'Amici, Nan Shen, Yongguo Yu, Alicia Hillert, Nastassja Himmelreich, Jürgen G Okun and Georg F Hoffmann performed the analysis, evaluated study results, participated in drafting and revising of the manuscript and read and approved the final version before submission.

## ETHICS STATEMENT

All procedures followed were in accordance with the ethical standards of the responsible committee on human experimentation (institutional and national) and with the Helsinki Declaration of 1975, as revised in 2000 (5). Informed consent was obtained from all patients for being included in the study. This article does not contain any studies with animal subjects performed by the any of the authors.
